# The Instinct of Appetite

**Published:** 1882-03

**Authors:** 


### THE INSTINCT OF APPETITE.

Chemical analysis and physiological research have established, beyond dispute, that every article of food and drink is composed of elements differing in quantity or quality. It is equally true that the various parts of the human frame are different in their composition, as the bone, the flesh, the nerve, the tendon, etc. But there is no element in the human body which is not found in some article of food or drink. A certain normal proportion of these elements, properly distributed, constitutes vigorous health, and forms a perfect body. If one of these elements be in excess, certain forms of disease manifest themselves; if there is not enough, some other malady affects the frame. When the blood contains less than its healthful amount of iron, it is poor, watery, and comparatively colorless; the muscles are flabby, the face pale, the eyes sunken, the whole body weak, the mind listless and sad. If the bones have not enough lime, they have no strength, are easily bent, and the patient is rickety; if there is too much lime, then the bones are brittle, and are broken by the slightest fall or unusual strain. The highest skill of the physician in these cases consists in determining the excess or deficit of any element, and in supplying such food or drug as will meet the case; when the medical attendant can not determine what is wanting nor furnish the supply, nature is often loud enough in her calls, through the tastes or appetites, to indicate very clearly what item of food or drink contains the needed elements; this is the "Instinct of Appetite." Chemistry is unable to say of but one article of human food, that it contains all the constituents necessary to supply the human body with every element requisite for its welfare, and that is pure milk, as supplied by the mother of the new being; but after the first years of life, the body demands new elements, in order to enable it to meet the duties which increasing age imposes; hence, nature dries up this spring, as being no longer adequate, and compels the search for other kinds of sustenance, showing that milk is a proper sole food for the young ones; and healthy grown persons who live upon it mainly will always become invalids.

All kinds of life, whether vegetable or animal, have within them a principle of preservation, as well as of perpetuity; were that not the case, all that breathes or grows would die; this principle or quality is common to man and beast, and all that springs from root or seed; it is named "Instinct." It is instinct which calls, by thirst, for water, when there is not fluid enough in the system. It is instinct which calls for food, by hunger, when a man is weak and needs renovation. It is curious and practically valuable as a means for the removal of disease, to notice the working of this instinct, for it seems to be almost possessed with a discriminating intelligence; certain it is, that standard medical publications give well-authenticated facts, showing, that following the cravings of the appetite, the animal instinct has accomplished far more than the physician's skill was able to do; has saved life in multitudes of cases, when science has done its best, but in vain.

About three years ago, the little daughter of a farmer on the Hudson river, had a fall, which induced a long, painful and dangerous illness, ending in blindness; medication availed nothing. By accident, a switch containing maple buds was placed in her hands, when she began to eat them, and called earnestly for more, and continued to eat them with avidity, improving, meanwhile, in her general health for some fifteen days or more, when this particular relish left her, and she called for candy, and, as in the case of the buds, ate nothing else for two weeks, when this also was dropped, a more natural taste returning with returning eyesight and usual health. This was instinct calling for those articles of food which contained the elements the want of which laid between disease and recovery.

A gentleman aged thirty-six, seemed to be in the last stages of consumptive disease, when he was seized with an uncontrollable desire for common table-salt; he spread it in thick layers over his meat, and over his bread and butter; he carried it in his vest-pocket, which was daily emptied by eating a pinch at a time. He regained his health, and remained well for years afterwards.

More recently, a case occurred in England of a child gradually declining in health, in spite of all that could be done by a remarkably shrewd and observant physician. On one of his visits, he found the father sipping a glass of toddy. The thought occurred to the doctor to offer some of it to the child, who took it with great satisfaction. The hint was improved; more was given, and more; and for two months this child of two years old lived almost wholly on whisky-toddy, when the desire declined, a more natural appetite returned, the health improving every hour, and was eventually entirely restored; but ever thereafter the child loathed the very smell or even sight of whisky-toddy.

A similar case is reported where a sick child took a pint of ale daily, and nothing else for many days, ultimately recovering, when the sight of an ale-bottle could not be endured. The child of a New-Yorker was supposed to be dying of the "summer complaint." As a last and desperate resort, it was hurried off to Rockaway in August, having the (usually considered fatal) hiccup when it started. Immediately on its arrival, on a cold, raw, chilly evening, about an hour after sundown, some fresh milk from the cow was instantly boiled and offered to it. It was with difficulty that the bowl could be withdrawn from its poor emaciated fingers. After an hour's interval more milk was given, and nothing else, for a number of days. That child is now one of the heartiest, healthiest girls in New-York!

In the cases above given, the children could not name their cravings; but accident threw in their way what the instincts required. Grown persons can express their cravings. There are many persons who can record, from their own personal experience, the beginning of a return to health, from gratifying some insatiate desire. The celebrated Professor Charles Caldwell was fond of relating in his lectures, that a young lady, abandoned to die, called for some pound-cake, which "science" would have pronounced a deadly dose; but as her case was considered hopeless, she was gratified, and recovered, living in good health afterwards. But in some forms of dyspepsia, to follow the cravings is to aggravate the disease, life is made intolerable, and suicide closes the scene. In low fevers, typhoid, yielding to the cravings is certain death.

To know when and how to follow the instinct of appetite, to gratify the cravings of nature, is of inestimable value. There is a rule which is always safe, and will save life in multitudes of cases, where the most skillfully "exhibited" drugs have been entirely unavailing. Partake at first of what nature seems to crave, in very small quantities; if no uncomfortable feeling follows, gradually increase the amount, until no more is called for. These suggestions and facts find confirmation in the large experience of that now beautiful and revered name, Florence Nightingale, whose memory will go down with blessing and honor side by side with that of the immortal John Howard to remotest time. She says: "I have seen, not by ones or tens, but by hundreds, cases where the stomach not only craves, but digests things which have never been laid down in any dietary for the sick, especially for the sick whose diseases were produced by bad food. Fruit, pickles, jams, gingerbread, fat of ham, of bacon, suet, cheese, buttermilk, etc., were administered freely, with happy results, simply because the sick *craved* them."

